# Forever young? The ethics of ongoing puberty suppression for non-binary adults

**DOI:** 10.1136/medethics-2019-106012

**Published:** 2020-07-24

**Authors:** Lauren Notini, Brian D Earp, Lynn Gillam, Rosalind J McDougall, Julian Savulescu, Michelle Telfer, Ken C Pang

**Affiliations:** 1 Melbourne Law School, University of Melbourne, Melbourne, Victoria, Australia; 2 Biomedical Ethics Research Group, Murdoch Children's Research Institute, Parkville, Victoria, Australia; 3 Yale-Hastings Program in Ethics and Health Policy, Yale University, New Haven, Connecticut, United States and The Hastings Center, Garrison, New York, United States; 4 The Oxford Uehiro Centre for Practical Ethics, University of Oxford, Oxford, United Kingdom; 5 Melbourne School of Population and Global Health, University of Melbourne, Melbourne, Victoria, Australia; 6 The Royal Children’s Hospital Melbourne, Parkville, Victoria, Australia; 7 University of Oxford, Oxford, United Kingdom; 8 University of Melbourne, Melbourne, Victoria, Australia; 9 Department of Adolescent Medicine, The Royal Children’s Hospital Melbourne, Parkville, Victoria, Australia; 10 Murdoch Children's Research Institute, Parkville, Victoria, Australia; 11 Department of Paediatrics, University of Melbourne, Melbourne, Victoria, Australia; 12 The Walter and Eliza Hall Institute of Medical Research, Melbourne, Victoria, Australia

**Keywords:** sexuality/gender, concept of health, clinical ethics, autonomy, philosophy of the health professions

## Abstract

In this article, we analyse the novel case of Phoenix, a non-binary adult requesting ongoing puberty suppression (OPS) to permanently prevent the development of secondary sex characteristics, as a way of affirming their gender identity. We argue that (1) the aim of OPS is consistent with the proper goals of medicine to promote well-being, and therefore could ethically be offered to non-binary adults in principle; (2) there are additional equity-based reasons to offer OPS to non-binary adults as a group; and (3) the ethical defensibility of facilitating individual requests for OPS from non-binary adults also depends on other relevant considerations, including the balance of potential benefits over harms for that specific patient, and whether the patient’s request is substantially autonomous. Although the broadly principlist ethical approach we take can be used to analyse other cases of non-binary adults requesting OPS apart from the case we evaluate, we highlight that the outcome will necessarily depend on the individual’s context and values. However, such clinical provision of OPS should ideally be within the context of a properly designed research study with long-term follow-up and open publication of results.

## Case study

Phoenix, 18, was assigned female at birth but has identified as gender non-binary (not entirely/exclusively male or female) since age 5. Phoenix uses they/them pronouns, has short hair and wears gender-neutral clothes. When Phoenix was 11, they began puberty and became extremely distressed by development of their breast buds and anxious about menstruation commencing soon. This prompted Phoenix and their parents to ask Phoenix’s paediatrician for puberty blockers to halt puberty and stop further pubertal development. At that time, Phoenix told their paediatrician they did not want to discontinue the use of such blockers in the future, as they did not want to go through any puberty. Given Phoenix’s severe distress, Phoenix’s paediatrician agreed puberty blockers should be given, but informed Phoenix and their parents he was not prepared to prescribe long-term puberty suppression, as this is riskier than short-term suppression. The paediatrician stated that, when Phoenix turned 16 and had a better sense of their gender identity, they would meet to discuss whether Phoenix wished to discontinue the puberty blockers and (1) revert to their endogenous (female) sex hormones or (2) commence testosterone.

When Phoenix turned 16, they informed their paediatrician that they did not want option (1) or (2). Rather, Phoenix was confident they would identify as non-binary for the rest of their life and wanted to stay on puberty blockers ‘forever’ to ensure their body remained in a ‘genderless’ state. Reluctantly, the paediatrician agreed to extend Phoenix’s time on blockers for another 2 years.

Recently, Phoenix entered the adult healthcare system and informed their new doctor that their desire to continue puberty suppression on an ongoing basis has not changed. Phoenix feels that remaining in an androgynous, peripubertal state is the only way that their body can truly reflect their non-binary gender identity. Phoenix, supported by their parents, has the financial means to pay for ongoing puberty suppression (OPS) (approximately $A5200 per year in Australia).

Phoenix does not have any underlying medical conditions which would specifically contraindicate hormonal intervention. Nevertheless, Phoenix’s new doctor feels that OPS is still too physically risky, especially with regard to bone health, and wonders if Phoenix has underlying psychological issues about not wanting to grow up.

Phoenix’s doctor refers Phoenix to a psychologist, who confirms that Phoenix continues to have significant distress about their body, similar in degree to that experienced by binary trans patients that the psychologist has seen. Phoenix has regular counselling with the psychologist, who judges that Phoenix’s distress is significant and enduring, and is not a symptom of an underlying psychopathology. The psychologist also reports that she does not see any signs that indicate Phoenix has a fear of growing up.

Phoenix tells the psychologist that they highly value having a body that matches their gender identity. Alternative options, including low-dose testosterone, menstrual suppression and future ‘top’ surgery, are unacceptable to Phoenix because they do not believe these alternatives would accurately reflect their non-binary gender identity.

Phoenix’s doctor and psychologist conduct a capacity assessment, and find Phoenix capable of consenting to OPS. The psychologist also verifies that Phoenix’s desire for OPS is long-standing, informed, voluntary and free from coercion.

Should Phoenix’s new doctor agree to prescribe puberty blockers for Phoenix to take on an ongoing basis?

## Introduction

In this paper, we identify and analyse the key ethical issues relevant to Phoenix’s case, a hypothetical yet realistic case based on clinical experience. Phoenix’s request raises novel ethical questions which have not previously been analysed. The ethical issues associated with puberty suppression for transgender or gender diverse individuals have only been explored in the context of a time-limited first step in a two-stage hormonal treatment pathway for gender binary (predominantly male or female) adolescents whose aim is ultimately to commence testosterone or oestrogen,^1^ or for non-binary adolescents.^2^ Here we examine whether puberty suppression should be offered as a stand-alone intervention for non-binary adults (18 or over) who started puberty suppression as adolescents, to affirm their gender identity (defined below).

Requests for ongoing puberty suppression (OPS) from non-binary adults raise three key ethical questions. First, does offering OPS to medically affirm non-binary gender identities align with the proper goals of medicine? Second, does equity require offering OPS to non-binary adults as a group? And, third, how should clinicians respond to individual requests for OPS from such adults? The first two questions are hurdle questions which must be addressed before establishing how clinicians should respond to individual requests. The third question relates to how clinicians should decide whether OPS is ethically justifiable in any given case.

We argue that it can be ethically justifiable for clinicians to offer OPS to non-binary adults as a group. Using a broadly principlist approach,^3^ we argue that whether OPS is ethically justified in any given case will depend on contingent and specific details that will vary with each non-binary adult who requests this intervention.^i^

## Background

Transgender (trans) individuals commonly seek medical interventions to affirm their gender identity. Trans individuals identify with a gender other than the one that corresponds to their sex-typed features observed at birth (often referred to as ‘birth-assigned sex’).^4^ Many, but not all, trans individuals experience gender dysphoria, a type of ‘discomfort or distress that is caused by a discrepancy between a person’s gender identity and that person’s sex assigned at birth (and the associated gender role and/or primary and secondary sex characteristics)’ (Coleman *et al*., p166)[4].

Gender identity has been formally conceptualised in different ways,^5^ but according to a standard clinical definition, it is ‘one’s internal, deeply held sense of gender’ (Hembree *et al.*, p3875)[6]. Gender identity is related to but distinct from the concept of gender, a wider category whose boundaries and grounding conditions (ie, for category membership) are often contested.^7 8^

Gender identity is often characterised as falling on a spectrum, with male or masculine at one end and female or feminine at the other end, and a range of different gender identities (including some non-binary gender identities) in between.^9^ Society is becoming increasingly aware and accepting of individuals such as Phoenix who identify as gender non-binary, and clinical guidelines explicitly acknowledge this group.^4 6 10^ These individuals do not identify as entirely or exclusively male or female, or potentially with any gender at all. They may identify with multiple genders, as neither male nor female, or as genderless.^11^ Such individuals may describe themselves as non-binary, genderqueer, gender-neutral, agender, or in other ways; we will use the umbrella term ‘non-binary’ in this paper. Studies report prevalence figures of 1.9%–4.6% for non-binary gender identities in the general population.^12 13^

Non-binary can be defined as a type of trans identity, as non-binary individuals identify with a gender other than the one that corresponds to their birth-assigned sex categorisation (or, indeed, identify with no gender at all). However, while non-binary is a type of trans identity according to this definition, not all non-binary individuals personally regard themselves as trans.^14^ None of the arguments we put forward in this paper (including those relating to the potential benefits of OPS for non-binary adults) rely on a non-binary person self-identifying as trans. Rather, the potential implications for welfare would depend on the individual and their circumstances.

### Puberty suppression for trans and gender diverse youth

Phoenix is requesting puberty blockers to prevent the development of unwanted and distressing secondary sex characteristics (breasts and menstruation). Puberty blockers (gonadotrophin-releasing hormone analogues, GnRHas) are typically administered via subcutaneous implant or injection given on a monthly, quarterly or yearly basis.^10^ Puberty suppression is often described in the literature as reversible; that is, if the young person discontinues puberty blockers, they will recommence the puberty consistent with the sex assigned to them at birth.^6^

Current guidelines recommend that puberty suppression should only be commenced when the young person reaches, at minimum, the second Tanner stage of puberty.^ii^ ^4 6 10^ There are several reasons for this recommended timing. First, waiting until Tanner stage 2 means that puberty suppression will not be given for a longer period of time than necessary, thereby helping to reduce the risks of side effects, such as low bone density.iii Second, the physical changes that occur during Tanner stage 2 are relatively minor (eg, development of small breast buds in birth-assigned females and slight testicular enlargement in birth-assigned males). Third, allowing some pubertal changes to occur may be beneficial. For example, in birth-assigned males, some degree of penile and scrotal growth is necessary if the young person wishes to keep certain surgical options (such as vaginoplasty) open for the future. Similarly, allowing some pubertal development may enable this group to store sperm for future reproductive purposes. Finally, it has been claimed that waiting until Tanner stage 2 can be useful in establishing a diagnosis of persistent gender dysphoria, as the adolescent has experienced some of the puberty consistent with the sex assigned to them at birth.^6^

## Does offering OPS to non-binary adults align with the proper goals of medicine?

Establishing whether Phoenix’s doctor should facilitate Phoenix’s request first requires analysing whether the aim of OPS is consistent with the proper goals of medicine. If not, it can be presumptively ruled out as a matter of ethical principle, at least as something which a *doctor* should do.

One way to clarify the ethical boundaries of medicine as a profession is to take a virtue ethics approach, which highlights what morally distinguishes one profession from others. A virtue ethics approach sees professional roles as being teleologically governed by the profession’s proper goals, or ‘the substantive good [the profession] undertakes to serve’ (Oakley & Cocking, p76)[15]. The general consensus in the literature is that medicine should serve the overarching goal of promoting patient health. ‘Good’ doctors will be committed to serving their patients’ health, and will practise in ways that demonstrate their commitment to this goal.^15^

What, then, is health? According to one dominant account, health is the absence of disease, defined as a condition that constitutes a deviation from statistically normal^iv^ species (‘species-typical’) functioning.^16^ Hence, it has typically been regarded as ethically defensible for medicine to aim at treating and preventing disease, but not at ‘enhancing’ normal functioning.^17^

Yet it has been argued that this ‘absence-of-disease’ account does not track what is morally significant about health, and thus fails to track what is virtuous about medical practice (ie, the proper goals of medicine).^18^ Hence, we employ a broader account of health as well-being, as per the World Health Organization (WHO), who equate health to ‘a state of complete physical, mental and social well-being and not merely the absence of disease or infirmity’ (pp 1–2).^19^ Some authors writing in bioethics call this the welfarist account, or the position that the proper goal of medicine is to promote not just health in the narrow physical sense, but health as overall well-being.^18^

Three main well-being theories have been put forward in the literature (for an overview, see Savulescu).^20^ The first, ‘hedonistic’ or ‘mental state’ theories, use mental states to define well-being.^21^ According to the simplest versions of these theories, the only inherently valuable mental state is pleasure or happiness and the only intrinsically bad mental state is pain or unhappiness. A hedonistic theory of well-being holds that it may be ethically defensible for a non-binary adult, such as Phoenix, to choose OPS if this provides them with pleasure or happiness (eg, feeling happier about their body).^20^

The second, ‘desire fulfilment’ theories, hold that well-being entails fulfilling one’s (sufficiently) freely formed and (sufficiently) informed desires. Desire fulfilment theories acknowledge that people have different values which may inform their desires. According to a desire fulfilment theory of well-being, it may be ethically defensible for Phoenix to choose OPS if they have a sufficiently free and informed desire to do so, and OPS is reasonably expected to fulfil this desire.^20^ The desire fulfilment theory of well-being involves an obvious connection between autonomy and well-being, in that promoting a person’s autonomy (through fulfilling their informed desires) would also promote their well-being.

The third, ‘objective list’ theories of well-being, hold that there are certain states or activities that are objectively good and can promote well-being, regardless of whether people desire them or whether they produce pleasurable mental states.^22^ Things that have been described as objective goods include individuality, independence, forming and maintaining meaningful relationships with others, having and rearing children, developing one’s talents, achieving worthwhile goals and gaining knowledge.^20 22^ According to an objective list theory of well-being, it may be ethically defensible for Phoenix to choose OPS if it may lead to objective goods for them (eg, achieving a goal or completing a project related to self-development, through bringing their body and gender identity into better alignment).

A composite theory of well-being, comprising aspects of each of the above theories (objective and subjective elements), is endorsed by many philosophers.^23 24^ This composite theory regards well-being as ‘engaging in objectively worthwhile activities which we desire and which provide us with pleasure’ (Savulescu, p23)[20]. A composite theory captures the important values contained within each of the above three theories, but avoids problems associated with adopting a single approach to well-being.^20 22^

We do not adopt a particular theory of well-being in this paper. Rather, we simply acknowledge that well-being is broader than physical health alone, and includes other components (eg, happiness, pleasure, life satisfaction).^21^ While promoting a person’s physical health usually promotes their well-being (eg, by allowing them to continue fulfilling their life plans), one’s well-being might sometimes be promoted at the expense of their physical health (eg, if one engages in a physically risky activity which is nevertheless of great value to them). This position, in our view, is consistent with the ethical principle of beneficence as framed by Beauchamp and Childress, which does not limit itself to physical benefits but rather considers welfare more broadly.^3^ In addition, this position underlines a number of interventions that doctors routinely—and seemingly justifiably—provide, such as contraception, abortion, assisted reproduction and some cosmetic surgery. The goal of these interventions is usually not to treat disease, but to promote patients’ broader well-being.

OPS could improve non-binary adults’ well-being on several grounds, including by leading to pleasure/happiness, fulfilling their informed desires and resulting in objective goods. For example, OPS could increase self-esteem and interpersonal functioning, in part by allowing the person to feel more comfortable in their body as they navigate the social world. As OPS may promote the well-being of non-binary adults in various ways, we argue that offering this intervention to this group, in principle, aligns with the proper goals of medicine—on a welfarist account of these goals—irrespective of which theory of well-being one adopts.

Some may question whether doctors have a duty to take any and all actions that are expected to improve aspects of a patient’s well-being (eg, self-esteem and interpersonal functioning). We argue that they do not have such a duty. This is because the goal of medicine is to promote patient well-being, using the *tools of medicine*.^v^ These tools of medicine include medication, equipment, training and diagnostic skills. We acknowledge that the proper realm of medicine will inevitably involve blurry edges, and it will not always be possible to draw clear boundaries. However, prescribing a regulated medication (here, puberty suppression) is clearly a medical activity which can only be carried out by registered medical practitioners. We therefore conclude that offering OPS as an option for non-binary adults falls within the proper goals of medicine.

## Does equity require offering OPS to non-binary adults?

As OPS could promote the well-being of some non-binary adults, it cannot be automatically ruled out through appeals to (violating) the proper goals of medicine. In this section, we argue that equity constitutes an additional ethical reason to offer this intervention to non-binary adults as a group.

The ethical principle of justice includes the notion of justice as equity (the Aristotelian maxim to ‘treat like cases alike’) and distributive justice (fair allocation of limited resources). While considerations regarding distributive justice are ethically important, we do not have sufficient space to discuss these in this paper; we focus only on justice as equity. We have deliberately highlighted in our hypothetical scenario that Phoenix can self-fund OPS, in order to sidestep distributive justice questions about who should fund this intervention (patients, patients’ insurance companies or the public healthcare system).^vi^ This allows us to focus on more fundamental ethical questions, including whether medicine should even offer OPS as an option for non-binary adults in the first place.

Offering gender-affirming interventions (including puberty suppression) to trans and gender diverse (TGD) people who have a *binary* gender identity is now commonplace in medicine, partly on the basis that such interventions aim to promote well-being by resulting in a physical appearance the patient regards as better aligning with their gender identity.^4^ This raises the question of why such interventions should not also be offered for similar reasons to *non-binary* TGD people as a matter of equity. Differential treatment may constitute unjust discrimination if no morally relevant differences exist between these groups. Presumptively, there is no inherent moral difference between binary and non-binary gender identities as such; both are ways of making sense of one’s self in relation to gender that could be undermined by incongruent bodily appearance, and in both cases medical intervention could be used to address the incongruence as a means to promote well-being. All else being equal, which gender identity a person has is morally irrelevant to determining whether they should be permitted to access gender-affirming interventions.

All else may not be equal, however. We acknowledge that there is potentially a difference in the type or degree of physical risks involved. While hormonal intervention involves physical risks for both binary and non-binary TGD individuals, these risks are more likely in the case of OPS, as we describe in the following section. This difference in risk of harm is morally salient, as it is highly relevant in determining whether an intervention is likely to promote or reduce an individual’s well-being, all things considered. However, we argue that potential harms (including the significance of physical risks) should be assessed in the context of the individual and their circumstances and values. OPS cannot be regarded as detrimental to well-being for all non-binary adults as a group. We suggest that, for some non-binary adults, the potential benefits of OPS may significantly outweigh the potential harms (including physical risks), meaning that their well-being would be enhanced by OPS.

## How should clinicians respond to individual requests for OPS from non-binary adults?

We have argued that OPS for non-binary adults is ethically justifiable in principle, for two reasons: alignment with the goals of medicine, and justice as equity in the Aristotelian sense. However, this is only the first step in deciding what is ethically appropriate for Phoenix. The next step is to make a fine-grained ethical evaluation of Phoenix’s particular situation.

The decision about whether it is ethically defensible for a clinician to facilitate an *individual* request for OPS to affirm a non-binary gender identity depends on several ethical considerations. Using a broadly principlist approach,^3^ these considerations include whether OPS is likely to promote the individual’s well-being (ie, whether the potential benefits are likely to outweigh the potential harms) and whether the individual’s request for OPS is substantially autonomous. We argue that both criteria are met in Phoenix’s case.

### OPS is likely to promote Phoenix’s well-being

The concept of well-being considers potential harms, in addition to potential benefits. If the potential benefits of OPS to Phoenix are greater than the potential harms, OPS is likely to promote Phoenix’s well-being. We describe the potential harms and benefits of OPS for Phoenix (summarised in table 1), and argue that the latter can reasonably be judged to outweigh the former.

**Table 1:** Potential harms and benefits of ongoing puberty suppression*

	Physical	Psychosocial	Cognitive
Potential harms	Localised reaction at injection/implant site (eg, swelling, redness, pain) and/or allergic reaction. Reduced bone density, increasing risk of osteoporosis and fractures. Impaired fertility. Impaired sexual functioning (which may include vaginal atrophy and pain during vaginal intercourse for birth-assigned females). Fusion of bone growth plates will be impaired, resulting in increased final height. Possible increased risk of developing hypertension, cardiovascular disease and metabolic disorders such as obesity, high cholesterol and type 2 diabetes.	Distress associated with any physical harms that eventuate. Could result in difficulty finding a romantic partner. Reduced libido. Later regret. Social stigma, which may have a negative impact on psychological functioning. Concerns about puberty suppression may lead to or increase attempted self-harm and/or suicide.	Potential negative impact on brain development.
Potential benefits	Prevents irreversible development of unwanted secondary sex characteristics. May prevent need for future gender-affirming surgeries.	Avoid distress associated with physical changes of puberty. Results in a physical appearance that better matches gender identity. Prevent/alleviate gender dysphoria and related psychosocial issues (eg, anxiety, depression). Improve overall psychosocial functioning and general mental health. Provides more time to consider gender identity and alternative options.	

Table assembled using information and arguments from ref 1 25 28–31 33 38 39 42 43 45.

*Some of the potential harms listed above could actually be considered benefits, and vice versa, depending on the patient’s values.

#### Potential harms of OPS

##### Physical harms

The potential physical harms of puberty suppression include risks related to the blockers themselves, such as the risk of a localised reaction (eg, swelling, redness, pain) at the implant or injection site or the risk of an allergic reaction. However, most of the physical risks of puberty blockers are due to their effect of blocking sex hormone production.

Exposure to sex hormones during puberty is important for bone strength. Consequently, puberty suppression is likely to reduce Phoenix’s bone density, increasing their osteoporosis and fracture risk.^25^ To reduce this risk, some clinicians have recommended limiting puberty suppression to 2 years.^26^ Statistics for the elderly population show that a 50-year-old birth-assigned female with a bone density in the lowest 2.5 percentile would have a 0.7%–1% risk of sustaining a hip fracture in the next 5–10 years, compared with a 0.1%–0.3% risk in a control with normal bone density.^27^ While no equivalent statistics exist for younger adults, if these statistics were true for Phoenix, then Phoenix would still have a very low absolute risk of fracture, even with lower bone density. Furthermore, bone-related risks may be assessed by using bone density scans to regularly monitor Phoenix’s bone density and hopefully mitigated byvii ensuring Phoenix exercises regularly and has adequate calcium and vitamin D levels.^10^

Another potential physical harm (which may also have psychosocial consequences) is impaired sexual function. Prepubertal genitalia will function quite differently compared with those that have gone through puberty, and OPS will likely impact on Phoenix’s sexual function (quite apart from any effects on libido). For example, vaginal lining is hormone responsive and it can be assumed that OPS will create long-term vaginal atrophy, which can in turn result in Phoenix experiencing pain during any vaginal intercourse.^28^

Puberty suppression would also impair Phoenix’s fertility. Phoenix’s eggs are unlikely to mature without sex hormones.^29^ While fertility preservation techniques exist (eg, ovarian tissue cryopreservation), these remain experimental, especially for individuals who have not gone through puberty,^10^ such as Phoenix. Current guidelines recommend the impact of puberty blockers on fertility and fertility preservation options be discussed with patients and families before beginning puberty suppression.^4 6 10^ Phoenix could cease puberty suppression and their eggs may subsequently mature to be able to produce a child. However, as discontinuing puberty blockers would result in unwanted feminisation and Phoenix losing their ‘non-binary’ physical appearance, this option may not be acceptable to Phoenix.

Other potential physical harms of OPS for Phoenix are likely to be similar to those of lifelong untreated hypogonadism, a condition in which individuals have lower than normal levels of testosterone and/or oestrogen. Insufficient oestrogen or testosterone in hypogonadal individuals impairs the fusion of bone growth plates, which can result in a taller-than-expected final height.^30^ Adults with untreated hypogonadism are also at increased risk of developing hypertension, cardiovascular disease and metabolic disorders including obesity, high cholesterol and type 2 diabetes.^31^ It is important to note that the relevance of these findings to our ethical analysis of Phoenix—who has never had significant sex hormones in their system from childhood—is unclear, as these studies focused on older men who simply had lower testosterone levels later in life. No increase in mortality has been shown for individuals with idiopathic hypogonadotropic hypogonadism (which arises in childhood),^32^ which may indicate the cardiovascular risks may not be significant in Phoenix’s case.

##### Psychosocial harms

In addition to potential distress associated with any physical harms that eventuate, there may be other psychosocial harms associated with OPS to make Phoenix look ‘more non-binary’. For example, Phoenix’s child-like body could mean they have trouble finding a romantic partner. A lack of sex hormones will also reduce Phoenix’s libido.^33^

Some may also be concerned that Phoenix may change their mind about either (1) their non-binary gender identity or (2) OPS in the future, and that this may result in significant psychological distress. We acknowledge the potential for regret is another relevant potential psychosocial harm to consider when evaluating the ethical justifiability of OPS for individual non-binary adults such as Phoenix. However, in other contexts in medicine, capable adults are permitted to make decisions they might later regret (eg, termination of pregnancy, cosmetic surgery); indeed, respecting ‘the right to be wrong’ is a crucial part of respecting autonomy (Diamond & Beh, p107)[34].

Puberty suppression is also highly reversible. If Phoenix later decides they wish to discontinue blockers, their endogenous female puberty will recommence, or they can commence testosterone therapy. Long-term puberty suppression may not be *completely* reversible, in the sense that it may result in long-term bone density changes that cannot be reversed entirely. Evidence suggests that, while the bone density of trans adolescents who have received puberty suppression significantly improves when these adolescents receive testosterone or oestrogen, it may not reach the bone density levels of age and birth-assigned sex-matched controls.^35^ As existing studies tend to have short follow-up periods, further research is required to ascertain whether reduction in bone density is a permanent or temporaryviii effect of puberty suppression.^36^ Nevertheless, long-term puberty suppression certainly appears more reversible than some elective interventions medicine currently offers (eg, tubal ligation, cosmetic surgery).

Phoenix may also experience social stigma in relation to their decision for OPS. Most Western societies revolve around the idea that there are only two genders, male and female, as evidenced, for example, by having separate ‘male’ and ‘female’ public restrooms^ix^ and clothing. Assisting individuals to ‘look’ more non-binary may lead to increased stigmatisation or ill treatment by others, as those others may not be able to readily categorise the individual as male or female. While such ill treatment would itself be morally wrong and Phoenix would not be morally responsible for bringing it about, it is something that should be discussed with Phoenix. There is a substantial body of literature on the problems associated with having people conform to restrictive social norms as a way of avoiding being treated badly by others; ultimately the people engaging in such treatment are obligated to change their behaviour in conjunction with broader social efforts to combat these problematic social norms^x^.^37^

Recently, concerns have been expressed that puberty suppression may lead to or increase attempts at self-harm and/or suicide. This is another potential psychosocial harm to consider in Phoenix’s case. Preliminary findings for 30 of 44 trans young people (11–15 years) taking puberty blockers (GnRHas) as part of a study on puberty suppression conducted by the Tavistock and Portman National Health Service Foundation Trust’s Gender Identity Development Service (GIDS) based in London and Leeds showed a statistically significant increase in the number of youth agreeing with the statement ‘I deliberately try to hurt or kill myself’ after 1 year on puberty blockers (with young people’s scores for this item as ‘sometimes’ increasing from 18.9% before taking blockers to 32.1% after taking blockers for 1 year).^38^ Some have expressed concern that these findings may suggest that puberty suppression causes or leads to an increase in attempted self-harm and/or suicide.^39^ However, these preliminary findings must be interpreted with caution. As described in the section below, TGD youth as a population—including those not taking puberty blockers—have been shown to experience extremely high rates of attempted self-harm (80%) and suicide (48%).^40^ It is possible that participants in the Tavistock study would still have reported attempts of self-harm and/or suicide, or more attempts, even if they were not taking puberty blockers. The Tavistock study also has several methodological limitations. For example, the sample size was small, and the GIDS acknowledged that this prevented drawing any meaningful conclusions from the data.^41^ Moreover, the study did not involve a control group of TGD young people not taking puberty blockers. It is therefore not possible to attribute the reported before/after increase in attempts at self-harm and/or suicide to the puberty blockers themselves.^41^

##### Cognitive harms

Some may be concerned that OPS may adversely affect Phoenix’s cognitive development, since adolescence is a time where executive functions and abstract thinking develop.^42^ Very few studies have investigated the effects of puberty suppression on brain development in TGD youth.^36^ One study found accuracy on a brain functioning assessment task was significantly lower in transgender females receiving puberty suppression compared with controls (females who were not transgender), but noted this may be a chance finding, given the small sample size (which included eight suppressed trans females and 24 cis-female controls).^43^ While we are unaware of any studies investigating the cognitive effects of puberty suppression on non-binary individuals, such as Phoenix, specifically, it is plausible that the risk of cognitive harm increases with increased duration of puberty suppression. The effect of long-term puberty suppression on cognitive development should be a priority for future study.^xi^ In addition, the psychological harms that often accompany being TGD (described below) may have a more negative impact on Phoenix’s brain than puberty suppression.

#### Potential benefits of OPS

##### Physical benefits

A potential physical benefit of OPS for Phoenix is that it prevents the development of irreversible and unwanted secondary sex characteristics. Puberty suppression is a more reversible option than going through puberty. If Phoenix goes through puberty, many of the resulting secondary sex characteristics are not reversible, but can only be altered with costly and complex gender-affirming surgeries (eg, mastectomy) in the future. Blockers can prevent the need for such surgeries.

##### Psychosocial benefits

The main potential benefits of Phoenix remaining on blockers long term are psychosocial. Before starting blockers, Phoenix experienced extreme gender dysphoria due to the development of their breast buds and anticipatory anxiety relating to menstruation. By preventing pubertal development and associated distress, OPS could maintain Phoenix’s body in a state they regard as reflecting their non-binary gender identity and prevent Phoenix’s gender dysphoria from returning. An increase in gender dysphoria could increase Phoenix’s risk of developing certain mental health conditions or trigger a mental health crisis. For example, a recent survey of 859 Australian TGD (including non-binary) young people found a significant proportion had been diagnosed with depression (75%), anxiety (72%), post-traumatic stress disorder (25%) or an eating disorder (23%). Furthermore, 80% of respondents reported having self-harmed and 48% attempted suicide.^40^

Some may question whether these mental health issues are the consequence or cause of a TGD identity; for example, whether some of these individuals have an underlying psychological condition that is leading both to self-harm and gender dysphoria. It is commonly assumed that the psychosocial harms associated with being TGD are due, generally, to some combination of gender dysphoria and the social stigma experienced by many TGD individuals.^1 44^ It is not known whether this general attribution is justified, as the causal pathways involved have not been adequately studied. However, in Phoenix’s case, the psychologist has judged that Phoenix’s distress is not a symptom of an underlying psychopathology. In addition, as described above, a decision to discontinue blockers poses evident risks to Phoenix’s psychological well-being that need to be considered. These risks will likely be exacerbated if the decision to cease is not Phoenix’s own voluntary decision.

Puberty suppression could also improve Phoenix’s overall psychosocial functioning and general mental health. Puberty suppression has been shown to have several psychosocial benefits for TGD youth, including reducing depression and overall emotional and/or behavioural problems and improving global functioning.^36 45^ There is some evidence to suggest that puberty suppression alone does not significantly affect symptoms of gender dysphoria.^45^ Chew and colleagues note that, in the case of binary trans adolescents, this is ‘not surprising, because GnRHas cannot be expected to lessen the dislike of existing physical sex characteristics associated with an individual’s birth-assigned sex nor satisfy their desire for the physical sex characteristics of their preferred gender’ (Chew *et al.*, p15)[36]. As Phoenix wishes for their body to appear ‘genderless’, and OPS can help achieve this, puberty suppression may be more likely to reduce Phoenix’s gender dysphoria compared with puberty suppression for binary trans young people.

Continued suppression would also give Phoenix more time to consider their gender identity and alternative options. Although Phoenix has been on puberty blockers for 7 years and continues to identify as non-binary, it is possible that their relation to or understanding of their gender identity may change and they may subsequently decide to cease puberty suppression.

#### Alternative options

Considering whether OPS would promote Phoenix’s well-being also requires identifying and comparing alternative options (including the option of no medical intervention) and their potential benefits and harms.^3^ If the potential benefits of OPS are equal to or outweigh the potential harms, Phoenix’s decision to pursue OPS could be regarded as reasonable. Conversely, Phoenix’s decision could be regarded as unreasonable if most or all the potential benefits associated with OPS can be obtained via an alternative, less risky option.^46^ It will therefore be important for Phoenix’s doctor to explore what Phoenix means by a ‘genderless’ body, and the nature of Phoenix’s fears and concerns.

One alternative might be for Phoenix to discontinue blockers and commence low-dose testosterone, which would cause some masculinisation (eg, facial hair growth, voice deepening), but less so than standard testosterone.^33^ This would likely result in a combination of characteristically masculine and feminine secondary sex traits (that would develop after ceasing blockers). Another alternative would be to specifically address Phoenix’s concerns about menstruation and breasts. In this scenario, Phoenix could discontinue the blockers, reverting to their endogenous sex hormones and recommencing female puberty, but then take medication (eg, continuous use of the contraceptive pill) for long-term menstrual suppression. In this case, Phoenix’s breasts would start to grow, but Phoenix could bind them and/or have a mastectomy to remove them. If Phoenix only had issues with breasts and menstruation, this alternative might be acceptable to Phoenix. However, as noted in the case scenario, Phoenix has rejected alternative options, on the basis that they do not regard these alternatives as resulting in a physical appearance that accurately reflects their non-binary gender identity.

In addition, these alternatives have their own risks of harm. They may lead to Phoenix’s gender dysphoria (and associated psychosocial harms) returning or worsening. Phoenix’s rejection of these alternative options suggests Phoenix is distressed about menstruation and breasts, and about *any* secondary sex characteristics (eg, wider hips) that would develop with these alternatives. By preventing the development of irreversible secondary sex characteristics, OPS could avoid gender dysphoria, even though this option has risks (eg, low bone density) that alternatives do not. Phoenix’s decision for OPS could therefore be regarded as a reasonable choice.

#### Weighing potential benefits and harms

The question of whether OPS will promote Phoenix’s well-being, all things considered, is not an ethically straightforward one to answer. Assessing the potential harms and potential benefits of OPS requires considering Phoenix’s goals and values. It also depends on taking seriously Phoenix’s understanding of what would make their life go well, and their views about the potential psychosocial benefits of OPS. Some of the potential harms described above seem more subjective in nature than others. For example, difficulty forming romantic relationships and infertility are presumably only harms if Phoenix wishes to have a romantic partner (and their preferred candidate(s) for a partner would be deterred by their appearance) and genetic children. If Phoenix does not wish for these, it is hard to see how these can properly be regarded as harms to Phoenix. Other potential harms, such as low bone density leading to increased fracture risk—which presumably no-one wants—seem more objective and less values dependent. However, Phoenix could weigh these potential physical harms alongside the potential psychosocial benefits and come to a sufficiently autonomous decision that having a body that they see as better matching their gender identity is more important than having normal bone density. Phoenix may prefer to live life with low bone density (and associated fracture risks) and an appearance they see as congruent with their gender identity versus having healthy bone density and appearance they see as incongruent. While we have highlighted that there is insufficient evidence to suggest that the potential harms of OPS are excessive for Phoenix, interventions for capable adults can still be ethically permissible even when risks of harm are high. If Phoenix’s conception of their own well-being is that their physical health is not as important as living in accordance with their gender identity (through bodily changes), that is a compelling reason in favour of OPS for Phoenix. In other areas of life and medicine, capable adults are permitted to make lifestyle choices or engage in certain activities they deem worthwhile, even though these may increase physical risks (eg, smoking, cosmetic surgery, risky professions).

While we have argued that OPS could plausibly promote Phoenix’s well-being, all things considered, there is genuine uncertainty that must be addressed. This uncertainty is not solely due to the value-laden disagreements described above. There is a lackxii of methodologically rigorousxiii evidence concerning the long-term outcomes of relatively short-duration puberty suppression (eg, 3–5 years) for TGD individuals in general,^36^ let alone ongoing, potentially permanent puberty suppression for non-binary individuals such as Phoenix. Many of the potential harms of OPS we have described are largely speculative or have been extrapolated from clinically similar, but not identical, patient populations, such as individuals with hypogonadism. This lack of evidence contributes to the ethical complexity around these decisions, and makes weighing potential harms and benefits challenging. More objective evidence is needed about the actual benefits and harms of this option; such evidence would make analyses of potential harms and benefits more comprehensive and promote more informed decision-making. While randomised placebo-controlled trials are regarded as the gold standard for clinical research, they are not feasible in this context.xiv Case reports may be the best we can hope for. Any prescription of OPS should ideally (providing appropriate consent has been obtained) be part of a methodologically rigorous and properly designed research study, with long-term systematic follow-up similar to that described in recently published long-term study protocols.^47^ Furthermore, we are mindful that some of the main potential benefits of OPS for someone like Phoenix are highly subjective in nature—for example, having their body align with their gender identity—and therefore difficult to ascribe objective value to (or measure in a scientifically meaningful way). Thus, even if there was concrete evidence that OPS is physically harmful, individuals such as Phoenix might still make a substantially autonomous decision to accept these potential harms to promote these other aspects of their well-being.

### Phoenix’s choice as substantially autonomous

Respect for autonomy involves respecting people’s rights to have and express their views, make their own choices and act based on their beliefs and values (assuming they do not unduly harm others by doing so).^3^ There are many different conceptions of autonomy. According to some authors, respect for autonomy involves more than merely allowing a patient to select their preferred course of action, free of coercion and ignorance, from available options disclosed to them. Rather, it requires that individuals understand, critically evaluate and reflect on their preferences and values, ascertain whether these are desirable and act (sufficiently) freely to implement these values.^48^

On such comprehensive accounts of autonomy, very few decisions can be deemed fully autonomous; such standards are therefore unrealistic.^3^ Given that full autonomy is arguably difficult for most people to achieve most of the time,^xv^ requiring that Phoenix’s request for OPS must be *fully* autonomous would be setting an unfairly high bar that is not set in other areas of healthcare. As Beauchamp and Childress have argued:

No theory of autonomy is acceptable if it presents an ideal beyond the reach of ordinary, competent agents and choosers…[t]o restrict adequate decision making by patients…to the ideal of fully or completely autonomous decision making strips their acts of any meaningful place in the practical world, where people’s actions are rarely, if ever, fully autonomous (Beauchamp & Childress, p104)[3].

On a more common account of autonomy that is arguably adopted in healthcare, Phoenix’s decision need only be *substantially* xvi autonomous. Phoenix’s decision can be deemed substantially autonomous if it is made intentionally, Phoenix adequately understands information material to the decision and sufficiently appreciates its relevance, and Phoenix is substantially free from constraint when making the decision. *Full* understanding or *complete* freedom from influence is not required.^3^ As noted in the case description at the beginning of this paper, Phoenix’s decision meets each of these criteria. Phoenix’s decision for OPS can still be substantially autonomous, even if Phoenix’s doctorxvii believes the potential harms outweigh the benefits.

While it is important to note that Phoenix’s decision for OPS appears voluntary at present, Phoenix’s doctor would also need to regularly confirm that Phoenix still wants OPS. Phoenix’s doctor should advise Phoenix that they can change their mind at any time. A decision for OPS is not necessarily equivalent to a decision to remain on blockers *forever*; rather, it is a decision to stay on blockers for an undefined time.

We have assumed that Phoenix’s clinicians have already sought to promote Phoenix’s autonomy by encouraging Phoenix to discuss and reflect on their wishes, goals and values regarding their body, assisting Phoenix to identify and articulate the beliefs and values underlying their decision for OPS, and helping Phoenix to choose the course of action that is most likely to realise their considered values.^48^ Phoenix emphasises the great value they place on their body matching their gender identity. OPS can realise this value, as it would prevent unwanted feminisation, which Phoenix regards as inconsistent with their non-binary gender identity.

We have argued that Phoenix’s decision for OPS could be regarded as substantially autonomous, even if it involves accepting risks to physical health, for the sake of achieving other goals which Phoenix regards as more valuable. It is not necessary to value physical health above all else for a decision to count as autonomous. Respecting Phoenix’s autonomy carries intrinsic value; but, as we noted earlier, also carries instrumental value if one adopts a desire fulfilment theory of well-being. Hence, there are double grounds to respect Phoenix’s autonomy in this case (doing so is intrinsically valuable, and part of promoting well-being).

## Conclusion

We have argued that it is ethically defensible in principle for clinicians to offer OPS to non-binary adults as a group, as OPS can promote patient well-being and is therefore consistent with the proper goals of medicine. We also highlighted that, as gender-affirming interventions are routinely offered to binary TGD individuals on well-being-promoting grounds, and there is presumptively no morally relevant difference between binary and non-binary gender identities as such, there is an additional equity-based argument for offering OPS to non-binary adults.

Using the resources of a standard, broadly principlist, approach to ethics in a clinical setting, we have analysed a case of a non-binary adult, Phoenix, requesting OPS. We have argued that OPS is ethically justifiable in Phoenix’s case, as the potential benefits are likely to outweigh the potential harms and capable adults have the right to take on risk of harm. We have also contended that Phoenix’s request can be regarded as substantially autonomous. Arguably, medicine is moving beyond its traditional, narrow goal of promoting health, biostatistically conceived. We have argued that Phoenix’s choice to remain on puberty suppression should therefore be facilitated by clinicians who have the means to safely do so. However, such clinical provision of OPS should ideally be within the context of a properly designed research study with long-term follow-up and open publication of results.

For each individual non-binary adult requesting OPS, providers and ethicists should use the same tools and concepts to analyse each case. However, as ethical analysis will be individualised to each patient, it is possible to come to a different conclusion for another patient. We are not arguing that every case of OPS for a non-binary adult is ethically justified, just that some can be, such as Phoenix’s case. In other words, in arguing that OPS is likely to be beneficial for Phoenix, all things considered, we are not arguing that OPS would be beneficial for all other non-binary adults who request it. Healthcare professionals need to consider each patient and their specific context. For example, while Phoenix has not been found to have any mental health conditions or psychosocial issues that may contraindicate OPS, other non-binary adults may be diagnosed with such conditions. It may be that another non-binary adult has significant mental health issues and that the healthcare professional reasonably believes that OPS would make them worse off from a mental health perspective. These potential contraindications would need to be taken into consideration, and may rule out OPS as an ethically justifiable option in particular cases.

This raises another pertinent ethical question: if OPS is not expected to benefit a particular non-binary adult (or, is expected to lead to significant harm), but the patient makes an autonomous decision to pursue it, does the patient have a right to OPS from an autonomy point of view? While we do not have scope to address this question here, we suggest that future ethical work addressing this question would be fruitful.

More ethical work is also needed regarding cases where other non-binary adults may request OPS for different reasons. For example, should a diagnosis of gender dysphoria be required for an individual to be eligible for puberty suppression? What if Phoenix *were* requesting OPS out of fear of becoming an adult—would facilitating their request be ethically defensible? What is the role of psychological comorbidity in decisions about whether or not to prescribe OPS? We also briefly highlighted, but largely set aside, questions relating to distributive justice and who should fund OPS in this paper. Further ethical analysis is needed on the topic of non-binary individuals who may benefit from OPS but who lack the ability to self-fund this intervention. We decided not to discuss the option of gonadectomy (removal of Phoenix’s ovaries), an alternative medical option that would also result in the absence of sex hormones and prevent unwanted feminisation. Gonadectomy would likely be cheaper than OPS, and distributive justice may favour it. If an ongoing lack of sex hormones due to OPS is ethically defensible in some cases, might gonadectomy to achieve this same outcome also be ethically defensible? This, however, is a topic for another paper.

## Footnotes

The question of whether a given intervention could meet Beauchamp and Childress’ four principles of biomedical ethics^3^ but be inconsistent with the proper goals of medicine, while theoretically interesting, is beyond the scope of this paper to address.

There are five Tanner stages of puberty, ranging from Tanner stage 1 (prepubertal) to Tanner stage 5 (adult), with Tanner stage 2 being the earliest stage of puberty.^10^

With the natural variation in timing of onset of puberty, starting puberty suppression at Tanner stage 1 could result in months or years of unnecessary treatment with accompanying risks.

For example, a trait may be considered statistically normal if it falls within 2 SDs of the mean for that trait in the population. Such cut-offs are usually somewhat arbitrary.

Non-medical professions may also aim to promote the well-being of their clients, using the different, *non-medical*, tools at their disposal.

The financial costs of puberty suppression ($A5200 per annum in Australia) would likely rule out OPS as a viable option for most patients if they must self-fund it.

While it should be possible to mitigate loss of bone density using these strategies, they are unlikely to prevent it in the absence of sex hormone. This is why, for example, some postmenopausal women choose hormone replacement therapy, since calcium, vitamin D and weight-bearing exercise can only do so much to ameliorate bone density loss.

As has been observed in cis-gender youth who undergo puberty suppression for precocious puberty.^49^

Although the number of all-gender public restrooms is increasing.

Such as promoting awareness of gender identities outside the binary.

Another response to concerns about OPS adversely affecting Phoenix’s cognition is to raise the example of the eunuchs of ancient China, who were usually castrated as children and then acted as court officials, wielding immense power in running the emperor’s affairs.^50^ Given this, it stands to reason that their cognitive functions were not necessarily significantly impaired by their lack of sex hormones, or at least not enough to prevent them from fulfilling their complex administrative roles. What their functioning would have been in the counterfactual condition where they had not been castrated is of course not knowable.

Chew *et al* conducted a systematic review of literature published over a 70-year period (1946–2017) and found only nine studies investigating the effects of puberty suppression on transgender youth.^36^

Methodological limitations of existing studies include being retrospective in nature, small sample sizes, no control group, no randomisation or blinding and a significant number of patients being lost to follow-up.^36^

It would be obvious which participants are receiving a placebo, as they would develop secondary sex characteristics while the treatment group would not. It would also presumably be difficult to recruit patients to such a trial.

For example, due to factors such as illness, nerves, distraction, lack of understanding or coercion, which may temporarily limit the person’s ability to decide.^3^

Beauchamp and Childress acknowledge that any attempt to draw a clear dividing line between ‘substantial’ and ‘insubstantial’ ‘may appear arbitrary’, and such criteria ‘are best addressed in a particular context’ (Beauchamp & Childress, p105)[3].

The question of whether Phoenix’s decision for OPS *objectively* involves unreasonable risk of harm is more challenging to answer. As we demonstrate, it is highly dependent on the individual’s case, circumstances and values.
